# Spectrum, dose, and duration of antibiotic exposure and risk of intensive care unit–acquired carbapenem-resistant gram-negative bacteria: a prospective cohort study

**DOI:** 10.1186/s13613-025-01605-1

**Published:** 2025-12-01

**Authors:** Zhihui Chen, Jing Wu, Xiangru Ye, Zhonghua Li, JingWang, Yueru Tian, Lei Zhou, Jie Ni, Jialin Jin, Wenhong Zhang

**Affiliations:** 1 Shanghai Institute of Infectious Disease and Biosecurity, Fudan University, Shanghai, China; 2 Department of Infectious Diseases, Shanghai Key Laboratory of Infectious Diseases and Biosafety Emergency Response, Shanghai Medical College, National Medical Center for Infectious Diseases, Huashan Hospital, Fudan University, 12 Wulumuqi Middle Road, Jing’an District, Shanghai, 200040 China; 3 Shanghai Sci-Tech Inno Center for Infection & Immunity, Shanghai, 200052 China; 4 Department of Neurocritical care unit, Shanghai Medical College, Huashan Hospital, Fudan University, Shanghai, China; 5 Department of Infection Control, Shanghai Medical College, Huashan Hospital, Fudan University, Shanghai, China; 6 Department of Laboratory Medicine, Shanghai Medical College, Huashan Hospital, Fudan University, Shanghai, China; 7 Department of Intensive Care Unit, Shanghai Medical College, Huashan Hospital, Fudan University, Shanghai, China

**Keywords:** Carbapenem-resistant gram-negative bacteria, Intensive care unit, Antibiotic exposure, Cross infection, Antibiotic stewardship, Risk factors.

## Abstract

**Background:**

While antibiotic exposure is a known key risk factor for acquiring Carbapenem-resistant Gram-negative bacteria (CR-GNB) in the ICU, the independent contributions and relative importance of its core dimensions—spectrum, dose, and duration—remain poorly understood. This study aimed to clarify these specific relationships to inform the optimization of antibiotic stewardship strategies.

**Methods:**

We prospectively enrolled consecutive adult patients admitted to 4 ICUs at a university hospital between March 2024 and January 2025. Patients were screened for CR-GNB upon admission and weekly. Antibiotic exposure was quantified by spectrum (Antibiotic Spectrum Index per antibiotic day [ASI]), dose (Defined Daily Doses [DDDs]), and duration (Length of Therapy [LOT]). The primary outcome was ICU-acquired CR-GNB. We used interval-censored Cox regression to assess associations. Restricted cubic splines were used to explore potential non-linear relationships, and relative importance analysis was performed to compare the impact of the exposure metrics.

**Results:**

Overall, 151 of 422 patients (35.8%) acquired CR-GNB during their ICU stay, with a median follow-up of 12.0 days (interquartile range, 8.0–17.0). ASI per antibiotic day was independently associated with an increased risk of ICU-acquired CR-GNB (adjusted Hazard Ratio [aHR] per 1-unit increase, 1.14; 95% Confidence Interval [CI] 1.09–1.19; *P* < 0.001), exhibiting a non-linear J-shaped relationship (*P* for nonlinearity = 0.027). In contrast, after full adjustment, DDDs were not significantly associated with CR-GNB acquisition (aHR per 1-unit increase, 0.89; 95% CI 0.69–1.15; *P* = 0.374), despite displaying a non-linear inverted U-shaped relationship (*P* for nonlinearity < 0.001). Similarly, LOT showed no significant independent association in the fully adjusted model (aHR per 1-day increase, 1.03; 95% CI 0.97–1.11; *P* = 0.214), although a non-linear trend suggested increasing risk with longer durations (*P* for nonlinearity < 0.001). Relative importance analysis identified ASI per antibiotic day as the most critical factor (*P* < 0.001), significantly outweighing both DDDs and LOT (*P* > 0.05).

**Conclusions:**

This study identifies ASI per antibiotic day as the principal independent risk factor for ICU-acquired CR-GNB, significantly outweighing the adjusted impact of DDDs or LOT. Therefore, prioritizing antibiotic spectrum optimization is crucial for stewardship strategies targeting CR-GNB prevention in the ICU.

**Trial registration:**

Chinese Clinical Trial Registry Identifier ChiCTR2400081352. Registered 28 February 2024.

**Supplementary Information:**

The online version contains supplementary material available at 10.1186/s13613-025-01605-1.

## Introduction

Carbapenem-resistant Gram-negative bacteria (CR-GNB), including carbapenem-resistant Enterobacterales (CRE), carbapenem-resistant Acinetobacter baumannii (CRAB), and carbapenem-resistant Pseudomonas aeruginosa (CRPA), have been identified by the WHO as high-priority bacterial pathogens [1]. Intensive care units (ICUs), as high-risk environments for critically ill patients, serve as centers for the emergence, spread, and infection of CR-GNB [2]. Acquisition rates for CR-GNB during ICU stay are reported to range from 2.6% to over 38.89% [3–6].

Antibiotic use is widely acknowledged as the primary driver selecting for and spreading antibiotic resistance [7]. This selection pressure is particularly amplified in the ICU environment due to the high intensity of antibiotic use. Extensive epidemiological studies consistently confirm that prior antibiotic exposure is a crucial risk factor for the acquisition of CR-GNB, in ICU patients [8]. To fully understand the impact of antibiotic exposure on CR-GNB acquisition risk, its key dimensions—antibacterial spectrum, dose, and treatment duration—must be considered [9]. Exposure to broad-spectrum antibiotics, especially carbapenems, is frequently reported as strongly associated with this increased risk [4, 10]. Evidence on dosing is complex, with some findings suggesting target concentrations for clinical cure may increase resistance selection [11]. For duration, studies indicate that prolonged exposure is associated with resistance acquisition [12–14].

However, significant gaps persist in comprehensively elucidating the independent contributions and relative importance of antibiotic exposure spectrum, dose, and duration on the risk of ICU-acquired CR-GNB. Understanding is limited by several key challenges. Existing research frequently evaluates only a single exposure dimension, hindering insight into their combined impact. Difficulties in handling time-dependent variables and confounding also complicate accurate risk estimation, introducing bias [15]. Furthermore, the precise shape of dose-response relationships between exposure and risk remains unclear. Clarifying these relationships is crucial for optimizing antibiotic stewardship.

This prospective cohort study aims to precisely quantify antibiotic exposure spectrum, dose, and duration to determine their independent associations and relative importance with the risk of ICU-acquired CR-GNB. Findings will inform more targeted antibiotic stewardship strategies in the ICU.

## Methods

### Study design, sites and participants

We conducted a prospective cohort study involving consecutive adults admitted to 4 ICUs at Huashan Hospital affiliated to Fudan University (Shanghai, China) between March 2024 and January 2025. Data were collected and managed using REDCap [16]. Upon ICU admission, all patients underwent rectal surveillance cultures for CR-GNB (including CRE, CRAB, and CRPA) within 48 h and weekly thereafter during their ICU stay. Clinical cultures from relevant sites (e.g., blood, urine, sputum) were obtained when infection was suspected per standard practice. Rectal CR-GNB surveillance culture methods are detailed in Methods S1. Infection control interventions during this period were described previously [17].

Patients were excluded if they were younger than 18 years, had CR-GNB detected before or within 48 h of ICU admission, or had fewer than 2 rectal surveillance cultures. For patients with multiple admissions during the study period, only the first was analyzed. Written informed consent was obtained from all participants. The study protocol was approved by the institutional ethics committee (KY2024-060) and registered with the Chinese Clinical Trial Registry (ChiCTR2400081352). We followed STROBE guidelines for reporting observational studies [18] (Table S1).

### Antibiotic exposure assessment

To comprehensively evaluate antibiotic exposure during the study period, we systematically assessed the dose, duration, and spectrum of antibiotic therapy administered to each patient from admission until ICU discharge or the date of first CR-GNB positive culture, whichever occurred first. Antibiotic exposure data were extracted from electronic medical records, encompassing antibiotic name, dose, frequency, and administration start and end dates. For dose quantification, we recorded daily administered doses and calculated defined daily doses (DDDs) according to World Health Organization (WHO) methodology [19]. Duration was quantified as length of therapy (LOT), defined as the number of days a patient received at least one systemic antibiotic agent [20]. Antibiotic spectrum was assessed using the Antibiotic Spectrum Index (ASI) per antibiotic day [21]. A complete list of all antibiotics, their detailed scoring matrix, final ASI scores, and the source for each score is provided in Table S2. Worked examples for antibiotic exposure metrics are provided in Methods S2.

### Outcome

Our outcome was ICU-acquired CR-GNB, defined as the first detection of CR-GNB (infection or colonization) occurring ≥ 48 h after ICU admission, in patients with negative CR-GNB clinical and/or surveillance cultures within 48 h of ICU admission. A positive CR-GNB result from either rectal surveillance or any clinical culture was considered indicative of CR-GNB acquisition, while CR-GNB status was determined to be negative only when rectal surveillance cultures were negative. Due to the interval sampling design, CR-GNB acquisition time was interval-censored between the last negative and first positive culture. After the first CR-GNB detection was recorded, subsequent detections for the same patient were not included in the analysis. Patients who remained CR-GNB negative throughout their ICU stay were right-censored at their last negative surveillance culture.

### Covariates

Covariates were categorized as time-fixed or time-dependent. Time-fixed covariates included: sociodemographic characteristics (age, female sex); medical history (emergency admission, prior hospitalization, prior antibiotic exposure, prior antibiotic count, ward transfers, prior ICU stays, surgery); comorbidities (renal failure, chronic pulmonary disease, liver disease, cerebrovascular disease, diabetes mellitus); Elixhauser Comorbidity Index [22]; and ICU Type (Neurosurgical, Multidisciplinary, Infectious Disease).

Time-dependent covariates, assessed for each screening interval, included: duration of invasive device use (central venous catheter, invasive mechanical ventilation, urinary catheter, nasogastric tube); colonization pressure [23]; and use of other drugs (PPI or H_2_RA, corticosteroids). For each screening period, colonization pressure was estimated as the average of the daily proportion of patients colonized/infected with CR-GNB. The daily proportion itself was calculated as the number of patients colonized or infected with CR-GNB divided by the total number of patients in the unit each day. Covariate definitions are detailed in Table S3.

### Statistical analysis

Categorical variables were summarized as counts (percentages) and continuous variables as means (Standard Deviation) or medians (interquartile range [IQR]). Baseline patient group characteristics were compared using t tests or Wilcoxon rank-sum tests for continuous data and χ² or Fisher exact tests for categorical data. No missing data were present for the variables included in the final analysis. Full details on sample size calculation are provided in Methods S3.

We used interval-censored Cox proportional hazards regression, incorporating time-dependent covariates and an Expectation-Maximization algorithm [24], to estimate the association between antibiotic exposure metrics and acquisition of CR-GNB. Three sequential models were fitted: Model 1 (unadjusted); Model 2 (adjusted for demographics, comorbidities, invasive procedures, clinical variables); and Model 3 (Model 2 plus the other 2 antibiotic exposure metrics). Antibiotic exposure was modeled continuously and as tertiles (lowest tertile reference).

Kaplan-Meier curves visualized unadjusted cumulative CR-GNB acquisition by exposure tertiles (compared using Wald tests). We reported unadjusted and adjusted hazard ratios (HRs) with 95% CIs. Potential non-linear associations were explored using restricted cubic splines with 4 knots [25]. Correlation analyses were performed between the three antibiotic exposure metrics to ensure no collinearity existed in the dataset (Figure S1). We assessed the proportional hazards assumption by testing the significance of covariate-time interaction terms using likelihood ratio tests (Table S4). Relative importance of risk factors was calculated using the partial χ² statistic minus predictor degrees of freedom [26].

To assess the robustness of our findings, we conducted several sensitivity analyses. We repeated the primary multivariable analysis: [1] restricting the outcome to only CRE acquisition, given the higher reliability of rectal swabs for CRE; [2] using a standard Cox proportional hazards model instead of interval censoring, to evaluate model dependency; [3] for CRAB/CRPA outcomes, adjusting the interval start time earlier in a worst-case scenario (assuming the preceding negative rectal swab was falsely negative) to address potential interval definition bias; [4] excluding patients with antibiotic use within 30 days prior to hospital admission, to minimize confounding from prior antibiotic exposure; and [5] stratifying the primary outcome into two subgroups (colonization only and clinical infection) to explore potential differences in risk factors (Methods S4).

Statistical analyses used STATA statistical software (version 18, STATA Corp) and R (version 4.3.2, R Foundation). Two-sided *P* < 0.05 was considered statistically significant.

## Results

### Patient characteristics

**Fig. 1:**
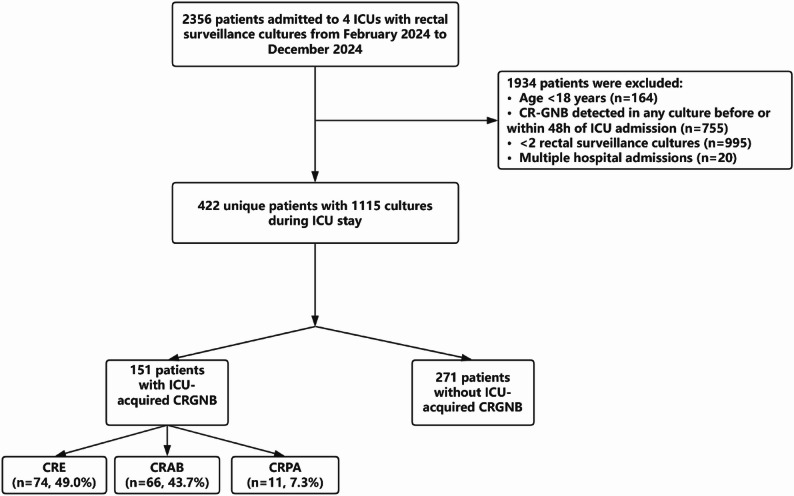
Flow Diagram of Study Population Selection. ICU intensive care unit, CR-GNB carbapenem-resistant Gram-negative bacteria, CRE carbapenem-resistant Enterobacterales, CRAB carbapenem-resistant Acinetobacter baumannii, CRPA carbapenem-resistant Pseudomonas aeruginosa

A total of 422 patients met the inclusion criteria and were included in the final analysis (Fig. 1). Baseline characteristics are presented in Table 1. The cohort had a median age of 59.0 years (interquartile range [IQR], 48.0–69.0) and included 170 female patients (40.3%). Over half of the admissions were emergencies (243 [57.6%]). The majority of patients were in the neurosurgical ICU (347 [82.2%]).

Regarding time-dependent covariates, the median duration of central venous catheter use was 4.0 days (IQR, 0.00–9.00), invasive mechanical ventilation was 2.0 days (IQR, 0.00–4.00), and urinary catheter was 3.0 days (IQR, 1.00–6.00). Other time-dependent variables are detailed in Table 1.

Median follow-up was 12.0 days (IQR, 8.00–17.00). ICU-acquired CR-GNB occurred in 151 patients (35.8%). The most frequent acquired organisms were carbapenem-resistant Enterobacterales (74 [49.0%]), carbapenem-resistant Acinetobacter baumannii (66 [43.7%]), and carbapenem-resistant Pseudomonas aeruginosa (11 [7.3%]) (Fig. 1).

**Table 1:** Baseline characteristics

Characteristics	All patients (*N* = 422)
Antibiotic exposure	
ASI per antibiotic day, median (IQR)	8.00 (6.00, 10.10)
DDDs, median (IQR)	1.02 (0.59, 1.61)
LOT, median (IQR), days	9.00 (6.00, 13.00)
Covariates	
Time-fixed	
Age, median (IQR), years	59.00 (48.00, 69.00 )
Female, No. (%)	170 (40.28)
Emergency admission, No. (%)	243 (57.58)
Hospitalization in the one month, No. (%)	26 (6.16)
Prior antibiotic exposure, No. (%)	83 (19.67)
Prior antibiotic count, median (IQR)	0.00 (0.00, 1.00 )
Ward transfers, No. (%)	164 (38.86)
Previous ICU episodes in current stay, No. (%)	38 (9.00)
Surgery, No. (%)	152 (36.02)
Renal failure, No. (%)	17 (4.03)
Chronic pulmonary disease, No. (%)	12 (2.84)
Liver disease, No. (%)	56 (13.27)
Cerebrovascular disease, No. (%)	167 (39.57)
Diabetes mellitus, No. (%)	62 (14.69)
Elixhauser Comorbidity Index, median (IQR), scores	2.00 (1.00, 8.00 )
SOFA score, median (IQR), scores	4.00 (2.00, 7.00 )
ICU Type, No. (%)	
Neurosurgical	347 (82.23)
Multidisciplinary	40 (9.48)
Infectious Disease	35 (8.29)
Time-dependent	
Central venous catheter, median (IQR), days	4.00 (0.00, 9.00 )
Invasive mechanical ventilation, median (IQR), days	2.00 (0.00, 4.00 )
Urinary catheter, median (IQR), days	3.00 (1.00, 6.00 )
Nasogastric tube, median (IQR), days	0.00 (0.00, 3.00 )
Colonization pressure, median (IQR), %	17.48 (10.94, 26.32 )
PPI or H_2_RA, median (IQR), days	8.00 (4.00, 12.00 )
Corticosteroids, median (IQR), days	2.00 (0.00, 8.00 )
Follow-up characteristics	
Median follow-up days, median (IQR)	12.00 (8.00, 17.00 )
Number of cultures, median (IQR)	2.00 (2.00, 3.00 )
Outcome	
ICU-acquired CR-GNB, No. (%)	151(35.78)

ASI antibiotic spectrum index, CR-GNB carbapenem-resistant Gram-negative bacteria, DDDs defined daily doses, LOT Length of therapy, H_2_RA histamine-2 receptor antagonist, ICU intensive care unit, IQR interquartile range, PPI proton pump inhibitor, SOFA Sequential Organ Failure Assessment. Values expressed as n (%) or median (IQR)

### ASI per antibiotic day and risk of ICU-acquired CR-GNB

The median ASI per antibiotic day in the study cohort was 8.00 (IQR, 6.00-10.10) (Table 1). We assessed the association between ASI per antibiotic day as a continuous variable and the risk of ICU-acquired CR-GNB (Table 2). In the unadjusted analysis, ASI per antibiotic day was significantly associated with risk (HR, 1.15; 95% CI 1.12–1.18; *P* < 0.001). The association remained significant after adjusting for patient baseline characteristics and time-dependent covariates (Model 2 h, 1.11; 95% CI 1.10–1.16; *P* < 0.001). After further adjustment for other antibiotic exposure metrics (DDDs and LOT), ASI per antibiotic day remained independently associated with the risk of ICU-acquired CR-GNB; each 1-unit increase in ASI was associated with a 13% increased risk (Model 3 h, 1.14; 95% CI 1.09–1.19; *P* < 0.001).

**Table 2:** Association between antibiotic exposure and risk of ICU-acquired CR-GNB

Antibiotic exposure metric	Model 1			Model 2		Model 3	
	HR (95%CI)	*P* value		HR (95%CI)	*P* value	HR (95%CI)	*P* value
ASI per antibiotic day							
per 1-unit increase	1.15 (1.12–1.18)		< 0.001	1.11 (1.10–1.16)	< 0.001	1.14 (1.09–1.19)	< 0.001
DDDs							
per 1-unit increase	1.64 (1.42–1.89)		< 0.001	1.34 (1.14–1.59)	< 0.001	0.89 (0.69–1.15)	0.374
LOT							
per 1-day increase	1.04 (1.00-1.08)		0.043	1.04 (0.98–1.11)	0.175	1.03 (0.97–1.11)	0.214

Model 1: Unadjusted

Model 2: Adjusted for age, sex, emergency admission, hospitalization in the one month, ward transfers, prior ICU stays, surgery, renal failure, chronic pulmonary disease, liver disease, cerebrovascular disease, diabetes mellitus, Elixhauser Comorbidity Index, ICU type (neurosurgical, multidisciplinary, infectious disease), central venous catheter use, invasive mechanical ventilation, urinary catheter, nasogastric tube, colonization pressure, PPI or H2RA use, SOFA, and corticosteroid use

Model 3: For each antibiotic exposure metric, adjusted for all variables in Model 2 plus the other two antibiotic exposure metrics

CR-GNB carbapenem-resistant Gram-negative bacteria, ICU intensive care unit, HR hazard ratio, CI confidence interval, ASI antibiotic spectrum index, DDDs defined daily doses, LOT length of therapy, PPI proton pump inhibitor, H_2_RA histamine-2 receptor antagonist SOFA Sequential Organ Failure Assessment

**Fig. 2:**
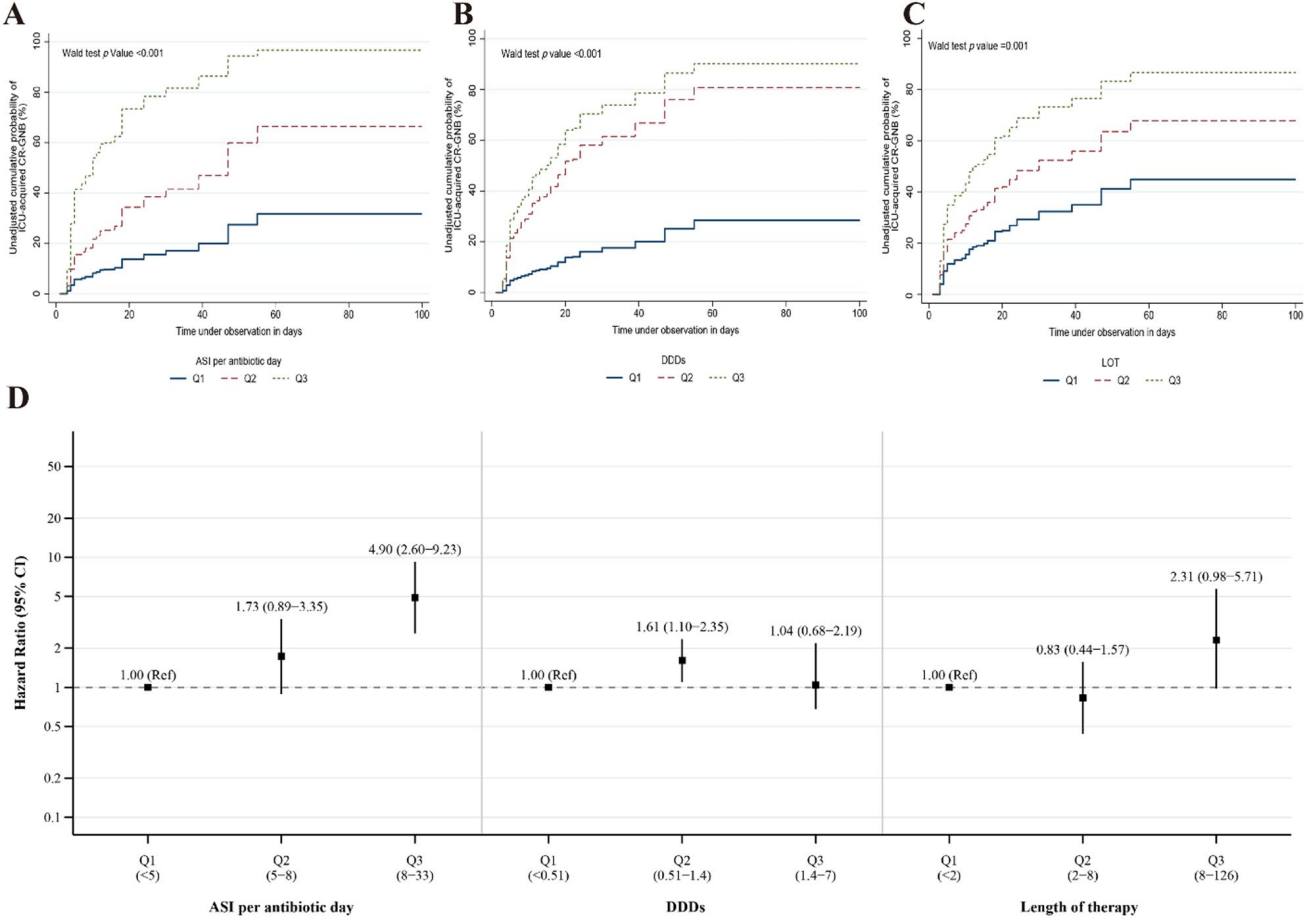
Association between antibiotic exposure metrics and acquisition of CR-GNB in ICU patients. **A** Unadjusted Kaplan-Meier curves showing cumulative incidence of ICU-acquired CR-GNB stratified by tertiles. **B** Unadjusted Kaplan-Meier curves for cumulative incidence of ICU-acquired CR-GNB by tertiles of DDDs. **C** Unadjusted Kaplan-Meier curves for cumulative incidence of ICU-acquired CR-GNB by tertiles of LOT. **D** Forest plot displaying adjusted hazard ratios with 95% confidence intervals for ICU-acquired CR-GNB by tertiles of each antibiotic exposure metric, with the lowest tertile (Q1) as reference. ASI Antibiotic Spectrum Index, DDDs Defined Daily Doses, LOT Length of Therapy, CR-GNB carbapenem-resistant Gram-negative bacteria, Q1 first (lowest) tertile, Q2 second (middle) tertile, Q3 third (highest) tertile

Analysis categorizing ASI per antibiotic day into tertiles further supported this finding. The cumulative incidence of ICU-acquired CR-GNB was significantly higher in groups with higher ASI levels (Wald test *P* < 0.001) (Fig. 2A). Tertile analysis showed that compared with low ASI levels (< 5), patients with moderate ASI levels [5–8] exhibited a trend toward increased CR-GNB acquisition risk (HR, 1.73 [95% CI 0.89–3.35]), while those with high ASI levels [8–33] had nearly 5-fold higher risk (HR, 4.90 [95% CI 2.60–9.23]).

**Fig. 3:**
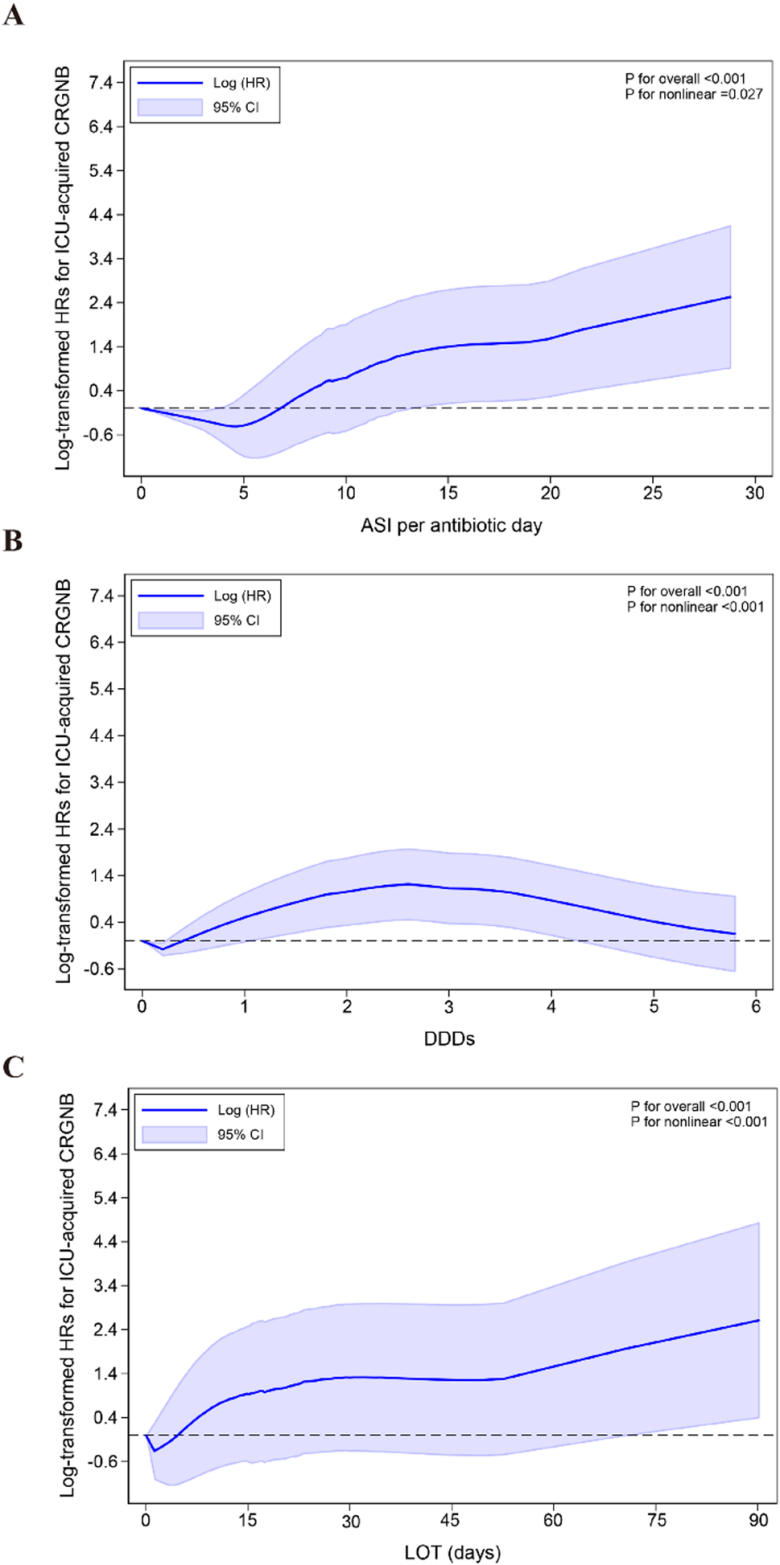
Non-linear associations between antibiotic exposure dimensions and risk of ICU-acquired CR-GNB. **A** Association between Antibiotic Spectrum Index (ASI) per antibiotic day and CR-GNB risk. **B** Association between Defined Daily Doses (DDDs) and CR-GNB risk. **C** Association between Length of Therapy (LOT) in days and CR-GNB risk. Restricted cubic spline models illustrating the non-linear relationships between antibiotic exposure metrics and log-transformed hazard ratios for ICU-acquired CR-GNB acquisition. Blue lines represent adjusted hazard ratios, with blue shaded areas indicating 95% confidence intervals. The dashed horizontal line at y = 0 represents HR = 1 (no effect). ASI Antibiotic Spectrum Index, DDDs Defined Daily Doses, LOT Length of Therapy, CR-GNB carbapenem-resistant Gram-negative bacteria, HR Hazard Ratio, CI Confidence Interval

Restricted cubic spline modeling revealed a significant non-linear, J-shaped association between ASI per antibiotic day and the risk of ICU-acquired CR-GNB (*P* for overall < 0.001; *P* for nonlinearity = 0.027) (Fig. 3A). The curve demonstrated a characteristic J-shaped pattern, with risk showing a slight decrease or plateau in the lower ASI range (approximately 0–5), followed by a steady upward trend.

### DDDs and risk of ICU-acquired CR-GNB

The median DDDs in the study cohort was 1.02 (IQR, 0.59–1.61) (Table 1). In the unadjusted analysis, DDDs as a continuous variable was significantly associated with the risk of ICU-acquired CR-GNB (HR, 1.64; 95% CI 1.42–1.89; *P* < 0.001). After adjusting for patient baseline characteristics and time-dependent covariates (Model 2), the association was attenuated but remained significant (HR, 1.34; 95% CI 1.14–1.59; *P* < 0.001). However, after further adjustment for other antibiotic exposure metrics (ASI and LOT) (Model 3), the association between DDDs and the risk of ICU-acquired CR-GNB was no longer statistically significant (HR, 0.89; 95% CI 0.69–1.15; *P* = 0.374).

Analysis categorizing DDDs into tertiles showed a significantly higher cumulative incidence of ICU-acquired CR-GNB in groups with higher DDDs levels compared to the lowest level group (Wald test *P* < 0.001). Tertile analysis revealed a non-monotonic relationship; compared with low DDDs levels (< 0.51), patients with moderate DDDs levels (0.51–1.4) exhibited a significantly increased CR-GNB acquisition risk (HR, 1.61 [95% CI 1.10–2.35]), while those with high DDDs levels (1.4-7) showed no significant elevation in risk (HR, 1.04 [95% CI 0.68–2.19]).

Restricted cubic spline modeling further suggested a non-linear association between DDDs and the risk of ICU-acquired CR-GNB, presenting as an inverted U-shaped relationship (*P* for nonlinear < 0.001, *P* for overall < 0.001) (Fig. 3B). Specifically, risk increased rapidly with increasing DDDs, peaked at approximately 2–3 DDDs, and subsequently decreased at higher DDDs levels.

### LOT and risk of ICU-acquired CR-GNB

The median LOT in the study cohort was 9.00 days (IQR, 6.00–13.00) (Table 1). In the unadjusted analysis, LOT as a continuous variable was associated with a statistically significant increased risk of ICU-acquired CR-GNB (HR, 1.04; 95% CI 1.00-1.08; *P* = 0.043). However, after adjusting for patient baseline characteristics and time-dependent covariates (Model 2), and further adjusting for ASI and DDDs (Model 3), the association between LOT and the risk of ICU-acquired CR-GNB was no longer statistically significant (Model 2 h, 1.04; 95% CI 0.98–1.11; *P* = 0.175; Model 3 h, 1.03; 95% CI 0.97–1.11; *P* = 0.214).

Analysis categorizing LOT into tertiles showed a significantly higher cumulative incidence of ICU-acquired CR-GNB in groups with longer LOT compared to the lowest group (Wald test *P* = 0.001). Compared with the shortest LOT tertile (< 2 days), patients with moderate LOT duration (2–8 days) showed no increased risk (HR, 0.83 [95% CI, 0.44–1.57]), while those with the longest LOT (8-126 days) exhibited a substantial elevation in risk that bordered on statistical significance (HR, 2.31 [95% CI, 0.98–5.71]).

Restricted cubic spline modeling suggested a non-linear association between LOT and the risk of ICU-acquired CR-GNB(*P* for nonlinear < 0.001, *P* for overall < 0.001) (Fig. 3C). The curve indicated that risk gradually increased with increasing LOT, with a more pronounced upward trend observed at longer durations of therapy.

### Relative importance of variables

**Fig. 4:**
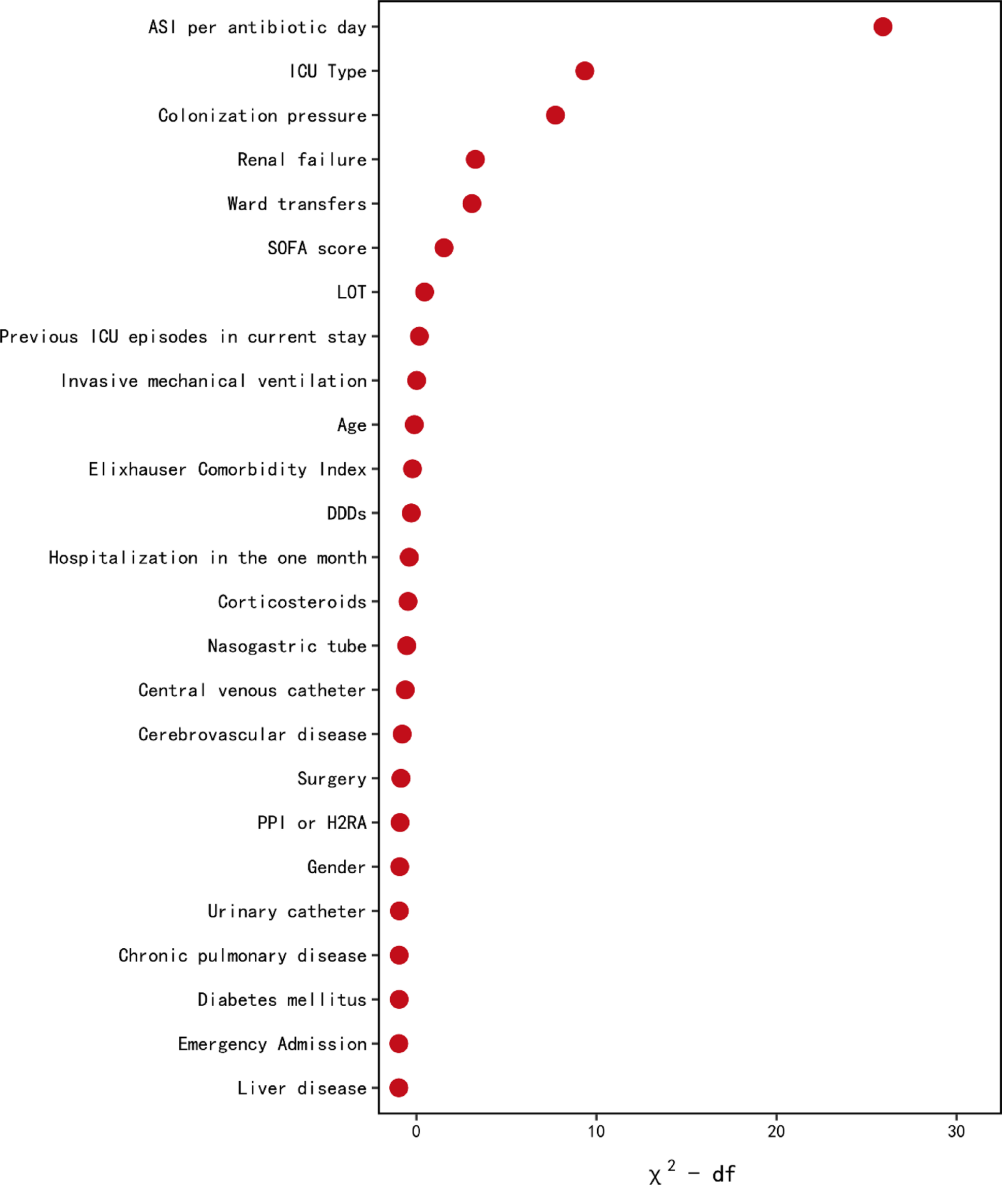
Relative importance of risk factors for ICU-acquired CR-GNB. The plot ranks variables based on their contribution to the fully adjusted interval-censored Cox regression model predicting the acquisition of ICU-acquired CR-GNB. Importance is quantified using the partial χ2 statistic minus the predictor’s degrees of freedom. ASI, Antibiotic Spectrum Index, DDDs Defined Daily Doses, H_2_RA histamine-2 receptor antagonist, ICU intensive care unit, LOT Length of Therapy, PPI proton pump inhibitor, SOFA Sequential Organ Failure Assessment

The ranking of variable importance in the constructed multivariable regression model for ICU-acquired CR-GNB is presented in Fig. 4 and Table S5. Results showed that ASI per antibiotic day had the highest relative importance among all variables included in the model (Importance = 25.92, *P* < 0.001). In comparison, other antibiotic exposure metrics, LOT (Importance = 0.46, *P* = 0.228) and DDDs (Importance = -0.28, *P* = 0.397), had lower relative importance and did not reach statistical significance.

### Sensitivity analyses

Sensitivity analyses confirmed the robustness of the primary findings (Table S6). Specifically, the association between ASI per antibiotic day and CR-GNB acquisition remained significant when restricting the outcome to CRE only (adjusted HR, 1.13 [95% CI, 1.08–1.18]), using a standard Cox model (adjusted HR, 1.11 [95% CI 1.07–1.15]), adjusting the CRAB/CRPA interval start time (adjusted HR, 1.07 [95% CI 1.03–1.22]), and excluding patients with prior 30-day antibiotic use (adjusted HR, 1.14 [95% CI 1.07–1.41]). Importantly, the association also held when stratifying the outcome into colonization only (adjusted HR, 1.15; 95% CI 1.07–1.26) and clinical infection only (adjusted HR, 1.10; 95% CI 1.02–1.33). In contrast, associations for DDDs and LOT with CR-GNB acquisition showed more variability across the sensitivity analyses.

## Discussion

Despite the recognition that antibiotic exposure is a critical risk factor for the acquisition of CR-GNB in ICU, the specific dimensions of antibiotic exposure most pivotal to this risk remain unclear. To address this critical gap, our prospective cohort study is the first to systematically evaluate and directly compare the relative importance of antibiotic spectrum, dose, and duration for the risk of ICU-acquired CR-GNB. We found that antibiotic spectrum, quantified by the ASI per antibiotic day, is the primary driver of ICU-acquired CR-GNB, demonstrating significantly greater importance than either antibiotic dose or treatment duration. Notably, all three dimensions of antibiotic exposure exhibited non-linear associations with CR-GNB acquisition—a threshold effect for spectrum, an inverted U-shaped relationship for dose, and accelerating risk with prolonged duration. These findings offer a crucial new perspective for antibiotic stewardship in the ICU setting, indicating that prioritizing the reduction of unnecessary broad-spectrum antibiotic use may be more critical for controlling CR-GNB dissemination than focusing solely on dose or duration.

Our principal finding indicates that the breadth of antibiotic spectrum, quantified by the ASI per antibiotic day, was the predominant and independent driver for CR-GNB in the ICU, surpassing the influence of DDDs and LOT. The biological underpinnings for this observation likely involve the potent selection pressure exerted by broad-spectrum agents, which not only eradicate susceptible competitors but critically disrupt the gut microbiome, thereby diminishing colonization resistance and facilitating CR-GNB establishment [27]. This finding contrasts with numerous studies focusing on exposure to specific high-risk antibiotic classes, such as carbapenems, or employing qualitative definitions of broad-spectrum exposure [28, 29]. The utilization of ASI provides a more granular, standardized, and quantitative assessment, enabling a better characterization of the cumulative pressure across diverse antibiotic classes known to contribute to CR-GNB selection [21, 30]. This study strongly suggests that antibiotic stewardship programs should prioritize optimizing spectrum selection—particularly through appropriate initial antibiotic choice guided by rapid diagnostic technologies, local resistance patterns, and clinical prediction tools to minimize unnecessary broad-spectrum exposure from treatment initiation [31, 32] —potentially yielding greater impact on CR-GNB acquisition than interventions solely focused on dose limitation or duration shortening. Furthermore, we identified a potential threshold effect, with a sharp, non-linear increase in CR-GNB acquisition risk observed when the ASI approached or exceeded approximately 5. This observation, distinct from potentially dose-response relationships suggested by population-level consumption analyses or some duration-specific studies [33, 34], may signify a critical level of cumulative spectrum pressure beyond which the risk escalates disproportionately, offering a potential quantitative target for risk stratification and focused stewardship interventions.

Contrasting with the pronounced, independent role of antibiotic spectrum, our study revealed that while DDDs showed a significant association in less adjusted models, this association lost statistical significance after additionally accounting for ASI per antibiotic day and LOT. This finding, potentially divergent from initial analyses or some previous reports emphasizing dose effect [35], suggests that the influence of this dimension might be substantially confounded by the choice of antibiotic spectrum or length of therapy. Despite its attenuated independent effect in the final model, dose exhibited a notable non-linear relationship with CR-GNB risk. The observed inverted U-shaped association for DDDs, where risk peaked at moderate doses (approximately 2–3 DDDs) before declining at higher levels, aligns with findings from several theoretical and experimental studies [36, 37]. Mechanistically, this pattern is often attributed to the mutant selection window (MSW) hypothesis [36]: moderate doses may fall within the MSW, inhibiting susceptible strains while providing competitive release and optimal conditions for resistant mutant amplification, whereas very high doses might suppress both susceptible and resistant populations more effectively, potentially by exceeding the mutant prevention concentration or reducing the bacterial load sufficiently to limit mutation supply. However, the substantial pharmacokinetic variability inherent in critically ill ICU patients—driven by pathophysiological changes such as increased volume of distribution from capillary leak and aggressive fluid resuscitation, as well as altered drug clearance from organ dysfunction or augmented renal clearance [38]—complicates the interpretation based solely on administered dose, as actual drug exposure often deviates significantly from prescribed doses. This imprecision, combined with the intrinsic nonlinearity linking antibiotic dose to resistance risk, may explain why a clear linear association for DDDs was not observed in the adjusted model.

Similarly, LOT did not demonstrate a statistically significant independent association in the final adjusted model, a trend toward increased risk with longer durations observed in spline analysis suggests a potential cumulative impact of prolonged exposure. Extended treatment inherently lengthens the period of selective pressure and microbiome disruption [39, 40], potentially increasing opportunities for bacterial adaptation or acquisition of resistance genes via horizontal transfer [41]. However, the association between duration and resistance emergence is increasingly recognized as complex and context-dependent, rather than a simple positive correlation. For instance, work by Mo et al. [42] indicated that while meta-analysis associated an additional day of antibiotic therapy with an approximate 7% absolute increase in the risk of resistant bacteria carriage, mathematical modeling further suggested this association is context-dependent. Specifically, shortening antibiotic treatment duration might increase resistance carriage when the administered antibiotic can effectively clear colonizing bacteria possessing a particular resistance phenotype. Therefore, our finding of a non-significant independent effect likely reflects both the dominant influence of antibiotic spectrum in our model and potentially specific characteristics of antibiotic use patterns within our ICU cohort. Nonetheless, given robust evidence supporting the clinical non-inferiority of shorter courses for many infections [43] and the established cumulative ecological impact of longer durations, optimizing treatment length remains an important component of antibiotic stewardship.

Notably, our analysis identified ICU Type as a powerful independent predictor for CR-GNB acquisition. While our model comprehensively adjusted for a wide range of patient-level characteristics, “ICU Type” likely serves as a proxy for unmeasured, unit-specific environmental factors, such as the local microbial ecology or distinct infection control practices. Therefore, including ICU Type in the model was crucial not only for highlighting its predictive power but also for helping to control for this potential source of confounding, strengthening the conclusion that antibiotic spectrum is the primary, direct driver.

This study possesses several strengths that bolster the validity of our findings. The prospective design minimized recall bias, while systematic surveillance and clinical cultures provided comprehensive detection of ICU-acquired CR-GNB. We employed precise, quantitative metrics for antibiotic exposure, including spectrum (ASI), dose (DDDs), and duration (LOT), moving beyond simpler categorical assessments. Furthermore, the application of interval-censored Cox regression allowed for appropriate handling of interval-censored outcomes and adjustment for time-dependent covariates, enhancing the robustness of our risk estimates.

However, certain limitations should be acknowledged. First, as a single-center study, our findings’ generalizability may be limited, although we included three different types of ICUs to enhance diversity. This is because the results may still reflect this hospital’s unique prescribing practices and local microbial ecology, thus limiting their extrapolation to other medical institutions. Second, rectal surveillance cultures might have lower sensitivity for detecting non-fermentative organisms like CRAB and CRPA compared to CRE [44]; however, our sensitivity analyses accounting for potential false negatives yielded consistent results. Third, despite comprehensive adjustment for numerous covariates including time-dependent variables, residual confounding from unmeasured factors cannot be entirely excluded. Lastly, our dose metric (DDDs) reflects prescribed therapy but does not account for the significant pharmacokinetic variability in critically ill patients, which affects true drug exposure.

## Conclusion

In conclusion, our study demonstrates that antibiotic spectrum is the primary determinant of ICU-acquired CR-GNB acquisition, outweighing dose and duration influences. These findings suggest that antimicrobial stewardship programs should prioritize spectrum optimization as the most critical intervention for combating carbapenem resistance.

## Supplementary Information

Supplementary Material 1.

## Data Availability

The datasets used and/or analysed during the current study are available from the corresponding author on reasonable request.

## Abbreviations

aHR: Adjusted Hazard Ratio
ASI: Antibiotic Spectrum Index
CI: Confidence Interval
CRAB: Carbapenem-resistant Acinetobacter baumannii
CRE: Carbapenem-resistant Enterobacterales
CR-GNB: Carbapenem-resistant Gram-negative bacteria
CRPA: Carbapenem-resistant Pseudomonas aeruginosa
DDDs: Defined Daily Doses
H_2_RA: Histamine-2 Receptor Antagonist
HR: Hazard Ratio
ICU: Intensive Care Unit
IQR: Interquartile Range
LOT: Length of Therapy
MSW: Mutant Selection Window
PPI: Proton Pump Inhibitor
REDCap: Research Electronic Data Capture
STROBE: Strengthening the Reporting of Observational Studies in Epidemiology
