# Supplementary material for: Spectrum, dose, and duration of antibiotic exposure and risk of intensive care unit–acquired carbapenem-resistant gram-negative bacteria: a prospective cohort study
Source: Ann Intensive Care. 2025 Dec 1;15:190. doi: 10.1186/s13613-025-01605-1 (PMC12665641; doi:10.1186/s13613-025-01605-1)
Supplement: Supplementary file 1 — Supplementary Material 1. [file 13613_2025_1605_MOESM1_ESM.docx]

**Spectrum, Dose, and Duration of Antibiotic Exposure and risk of Intensive Care Unit–Acquired Carbapenem-resistant Gram-negative Bacteria: A Prospective Cohort Study**

Zhihui Chen, PhD^1,2^ ^*^; Jing Wu, PhD^2*^; Xiangru Ye, MD, PhD^4*^; Zhonghua Li, MD, PhD^2^; JingWang, MD, PhD^5^; Yueru Tian^6^, PhD; Lei Zhou, MSN^2^; Jie Ni, MSN^7^, Jialin Jin, MD, PhD^2†^; Wenhong Zhang, MD, PhD^1,2,3†^

^*^These authors contributed equally as co-first authors.

^†^These authors contributed equally as co-corresponding authors.

Author Affiliations:

^1^Shanghai Institute of Infectious Disease and Biosecurity, Fudan University, China

^2^Department of Infectious Diseases, Shanghai Key Laboratory of Infectious Diseases and Biosafety Emergency Response, National Medical Center for Infectious Diseases, Huashan Hospital, Shanghai Medical College, Fudan University, China

^3^Shanghai Sci-Tech Inno Center for Infection & Immunity, Shanghai, 200052, China

^4^Department of Neurocritical care unit, Huashan Hospital, Shanghai Medical College, Fudan University, Shanghai, China

^5^Department of Infection Control, Huashan Hospital, Shanghai Medical College, Fudan University, Shanghai, China

^6^Department of Laboratory Medicine, Huashan Hospital, Shanghai Medical College, Fudan University, Shanghai, China

^7^Department of Intensive Care Unit, Huashan Hospital, Shanghai Medical College, Fudan University, Shanghai, China

**Methods S1.** Active Screening and Identification Protocol

**Methods S2.** Worked Examples for Antibiotic Exposure Metrics

**Methods S3.** Sample Size Calculation

**Methods S4.** Definitions for Colonization and Clinical Infection

**Table S1.** STROBE checklist

**Table S2.** Antibiotic spectrum index matrix

**Table S3.** Covariate definitions

**Table S4.** Proportional hazards assumption testing results

**Table S5.** Relative importance of variables

**Table S6.** Sensitivity Analyses

**Figure S1.** Correlation matrix of antibiotic exposure metrics.

**Methods S1.** Active Screening and Identification Protocol

Active surveillance targeted CR-GNB (including CRE, CRPA, and CRAB) using rectal swabs. Swabs were collected by inserting a sterile saline-moistened cotton swab 4-5 cm into the anus to obtain visible fecal matter. Initial screening occurred within 48 hours of ICU admission, followed by weekly surveillance. For ICU readmissions >48 hours after discharge, patients were screened as new admissions; otherwise, the prior schedule continued. A standardized protocol was used for CR-GNB identification: (1) Specimens were inoculated within 2 hours into 2 mL Tryptic Soy Broth (TSB) with 2 mg/L meropenem and incubated overnight (35°C, CO₂). (2) A 10 μL aliquot of the broth was subcultured onto China Blue Lactose Agar (CBLA) plates and incubated overnight (35°C, CO₂). (3) Typical colonies were subcultured onto Mueller-Hinton agar for disk diffusion testing (meropenem, imipenem, ertapenem) per CLSI criteria. (4) Confirmed carbapenem-resistant isolates underwent MALDI-TOF MS for species identification and were preserved.

**Methods S2.** Worked Examples for Antibiotic Exposure Metrics

We quantified antibiotic spectrum per calendar ICU day. For each day, every administered agent was assigned an ASI score according to Table S2. For combination therapy, the daily ASI equals the sum of the agent-level scores (days without systemic antibiotics have a daily ASI of 0). We then aggregated daily values into the exposure metric used in the main analysis: ASI per antibiotic-day, defined as the sum of daily ASI across the observation window divided by the number of antibiotic days (days with ≥1 systemic antibiotic). DDDs were averaged across antibiotic days (summing agent-level DDDs per day), and LOT counted calendar days with any systemic antibiotic. Worked example:

Days 1–3: vancomycin + piperacillin–tazobactam; Day 4: vancomycin only (piperacillin–tazobactam discontinued); Days 5–7: ceftriaxone only (vancomycin discontinued).

- ASI calculation. We sum daily ASI values for all concurrent antibiotics, then average over antibiotic days. Using the scores in Table S2 (illustrative values shown here: vancomycin = 5; piperacillin–tazobactam = 8; ceftriaxone = 5), daily ASI is: Days 1–3 = 13 each; Day 4 = 5; Days 5–7 = 5 each. Total ASI-days = (13×3) + (5×1) + (5×3) = 59. ASI per antibiotic day = 59 / 7 = 8.43.
- DDDs calculation. Cumulative DDDs for each agent are computed across the interval and then summed: vancomycin (Days 1–4) 1.0 DDD/day × 4 = 4.0; piperacillin–tazobactam (Days 1–3) 1.5 × 3 = 4.5; ceftriaxone (Days 5–7) 1.0 × 3 = 3.0. Total DDDs = 11.5; DDDs per antibiotic day = 11.5 / 7 = 1.64.
- LOT calculation. LOT counts calendar days with ≥1 systemic antibiotic. In this example, LOT = 7.

**Methods S3.** Sample Size Calculation

The statistical power of our study was formally validated through post-hoc sample size calculations using stpower cox in Stata. We used parameters derived directly from our final dataset, including observed hazard ratios (ASI: 1.14; DDDs: 0.89; LOT: 1.03) and standard deviations (ASI: 3.0; DDDs: 1.5; LOT: 5.2). With conventional statistical parameters (power=80%, α=0.05), the minimum required sample sizes were calculated to be 51, 257, and 333 participants for ASI, DDDs, and LOT, respectively. Our achieved sample size of 422 participants provided robust statistical power for all three primary exposure metrics (ASI: 100%; DDDs: 94.9%; LOT: 88.4%), confirming the study was adequately powered. While right-censored methods were used as an approximation due to the lack of established methods for interval-censored data, the high achieved power provides strong confidence in our statistical conclusions.

**Methods S4.** Definitions for Colonization and Clinical Infection

A patient was considered to have a clinical infection if the CR-GNB was isolated from a sterile body site (e.g., blood, cerebrospinal fluid, pleural fluid), or if a positive culture from a non-sterile site met established clinical criteria. Specifically, these criteria included meeting the American Thoracic Society (ATS) and Infectious Diseases Society of America (IDSA) guidelines for respiratory tract infections, or the U.S. Centers for Disease Control and Prevention (CDC) National Healthcare Safety Network (NHSN) definitions for urinary tract and surgical site infections. Furthermore, a non-surgical wound culture was classified as an infection only if it was documented as such by the attending physician and accompanied by evidence of a systemic inflammatory response (defined as an abnormal white blood cell count or body temperature). Any new positive CR-GNB culture that did not meet these criteria for clinical infection was classified as colonization.

**Table S1. STROBE checklist**

|  | Item No. | Recommendation | Location. |
| --- | --- | --- | --- |
| **Title and abstract** | 1 | (*a*) Indicate the study’s design with a commonly used term in the title or the abstract | Title, Abstract |
|  |  | (*b*) Provide in the abstract an informative and balanced summary of what was done and what was found | Abstract (Background, Methods, Results, Conclusions sections) |
| Introduction | | | |
| Background/rationale | 2 | Explain the scientific background and rationale for the investigation being reported | Introduction paragraphs 1-3 |
| Objectives | 3 | State specific objectives, including any prespecified hypotheses | Introduction final paragraph |
| Methods | | | |
| Study design | 4 | Present key elements of study design early in the paper | Methods, paragraph 1 |
| Setting | 5 | Describe the setting, locations, and relevant dates, including periods of recruitment, exposure, follow-up, and data collection | Methods, paragraph 1 |
| Participants | 6 | (*a*) *Cohort study*—Give the eligibility criteria, and the sources and methods of selection of participants. Describe methods of follow-up  *Case-control study*—Give the eligibility criteria, and the sources and methods of case ascertainment and control selection. Give the rationale for the choice of cases and controls  *Cross-sectional study*—Give the eligibility criteria, and the sources and methods of selection of participants | Methods, paragraphs 1-2 |
|  |  | (*b*) *Cohort study*—For matched studies, give matching criteria and number of exposed and unexposed  *Case-control study*—For matched studies, give matching criteria and the number of controls per case |  |
| Variables | 7 | Clearly define all outcomes, exposures, predictors, potential confounders, and effect modifiers. Give diagnostic criteria, if applicable | Methods, "Antibiotic Exposure Assessment" and "Outcome" sections; "Covariates" section |
| Data sources/ measurement | 8* | For each variable of interest, give sources of data and details of methods of assessment (measurement). Describe comparability of assessment methods if there is more than one group | Methods, "Antibiotic Exposure Assessment" section; Table S2; Table S3 |
| Bias | 9 | Describe any efforts to address potential sources of bias | Statistical analysis section |
| Study size | 10 | Explain how the study size was arrived at | Statistical analysis section；Methods S3 |
| Quantitative variables | 11 | Explain how quantitative variables were handled in the analyses. If applicable, describe which groupings were chosen and why | Statistical analysis section |
| Statistical methods | 12 | (*a*) Describe all statistical methods, including those used to control for confounding | Statistical analysis section |
|  |  | (*b*) Describe any methods used to examine subgroups and interactions | Statistical analysis section |
|  |  | (*c*) Explain how missing data were addressed | Statistical analysis section |
|  |  | (*d*) *Cohort study*—If applicable, explain how loss to follow-up was addressed  *Case-control study*—If applicable, explain how matching of cases and controls was addressed  *Cross-sectional study*—If applicable, describe analytical methods taking account of sampling strategy | Statistical analysis section |
|  |  | (*e*) Describe any sensitivity analyses | Statistical analysis section |
| Results | | | |
| Participants | 13* | (a) Report numbers of individuals at each stage of study—eg numbers potentially eligible, examined for eligibility, confirmed eligible, included in the study, completing follow-up, and analysed | Results, paragraph 1; Figure 1 |
|  |  | (b) Give reasons for non-participation at each stage | Figure 1 |
|  |  | (c) Consider use of a flow diagram | Figure 1 |
| Descriptive data | 14* | (a) Give characteristics of study participants (eg demographic, clinical, social) and information on exposures and potential confounders | Table 1; Results, paragraphs 1-2 |
|  |  | (b) Indicate number of participants with missing data for each variable of interest | Not explicitly reported |
|  |  | (c) *Cohort study*—Summarise follow-up time (eg, average and total amount) | Results, paragraph 3: "Median follow-up was 12.0 days (IQR, 8.00-17.00)" |
| Outcome data | 15* | *Cohort study*—Report numbers of outcome events or summary measures over time | Results, paragraph 3; Figure 1 |
|  |  | *Case-control study—*Report numbers in each exposure category, or summary measures of exposure |  |
|  |  | *Cross-sectional study—*Report numbers of outcome events or summary measures |  |
| Main results | 16 | (*a*) Give unadjusted estimates and, if applicable, confounder-adjusted estimates and their precision (eg, 95% confidence interval). Make clear which confounders were adjusted for and why they were included | Table 2; Results sections on ASI, DDDs, and LOT |
|  |  | (*b*) Report category boundaries when continuous variables were categorized | Results (tertile analyses in ASI, DDDs, and LOT sections) |
|  |  | (*c*) If relevant, consider translating estimates of relative risk into absolute risk for a meaningful time period | Not reported |
| Other analyses | 17 | Report other analyses done—eg analyses of subgroups and interactions, and sensitivity analyses | Results, "Sensitivity Analyses" section; Table S6 |
| Discussion | | | |
| Key results | 18 | Summarise key results with reference to study objectives | Discussion, paragraphs 1-2 |
| Limitations | 19 | Discuss limitations of the study, taking into account sources of potential bias or imprecision. Discuss both direction and magnitude of any potential bias | Discussion, final paragraph |
| Interpretation | 20 | Give a cautious overall interpretation of results considering objectives, limitations, multiplicity of analyses, results from similar studies, and other relevant evidence | Discussion sections |
| Generalisability | 21 | Discuss the generalisability (external validity) of the study results | Discussion, final paragraph |
| Other information | |  | |
| Funding | 22 | Give the source of funding and the role of the funders for the present study and, if applicable, for the original study on which the present article is based | Funding |

*Give information separately for cases and controls in case-control studies and, if applicable, for exposed and unexposed groups in cohort and cross-sectional studies.

**Note:** An Explanation and Elaboration article discusses each checklist item and gives methodological background and published examples of transparent reporting. The STROBE checklist is best used in conjunction with this article (freely available on the Web sites of PLoS Medicine at http://www.plosmedicine.org/, Annals of Internal Medicine at http://www.annals.org/, and Epidemiology at http://www.epidem.com/). Information on the STROBE Initiative is available at www.strobe-statement.org.

**Table S2. Antibiotic spectrum index matrix**

| Drug | MSSA | Enterococcus | Anaerobes | Bacteroides fragilis | Morxella/Haemophilus flu | Escherichia coli/ Klebsiella | Enterobacter/ Serriatia/ Citrobacter | ESBL | Pseudomonas | MRSA | Penicillin- resistant pneumonae | VRE | Atypical | MDRO | Antibiotic Spectrum Index | Source |
| --- | --- | --- | --- | --- | --- | --- | --- | --- | --- | --- | --- | --- | --- | --- | --- | --- |
| Dicloxacillin | 1 | 0 | 0 | 0 | 0 | 0 | 0 | 0 | 0 | 0 | 0 | 0 | 0 | 0 | 1 | Original Literature |
| Oxacillin | 1 | 0 | 0 | 0 | 0 | 0 | 0 | 0 | 0 | 0 | 0 | 0 | 0 | 0 | 1 | Original Literature |
| Amoxicillin | 0 | 1 | 0 | 0 | 0 | 1 | 0 | 0 | 0 | 0 | 0 | 0 | 0 | 0 | 2 | Original Literature |
| Ampicillin | 0 | 1 | 0 | 0 | 0 | 1 | 0 | 0 | 0 | 0 | 0 | 0 | 0 | 0 | 2 | Original Literature |
| Cephalexin | 1 | 0 | 0 | 0 | 0 | 1 | 0 | 0 | 0 | 0 | 0 | 0 | 0 | 0 | 2 | Original Literature |
| Erythromycin | 0 | 0 | 0 | 0 | 1 | 0 | 0 | 0 | 0 | 0 | 0 | 0 | 1 | 0 | 2 | Original Literature |
| Metronidazole | 0 | 0 | 1 | 1 | 0 | 0 | 0 | 0 | 0 | 0 | 0 | 0 | 0 | 0 | 2 | Original Literature |
| Penicillin | 0 | 1 | 1 | 0 | 0 | 0 | 0 | 0 | 0 | 0 | 0 | 0 | 0 | 0 | 2 | Original Literature |
| Aztreonam | 0 | 0 | 0 | 0 | 1 | 1 | 0 | 0 | 1 | 0 | 0 | 0 | 0 | 0 | 3 | Original Literature |
| Cefazolin | 1 | 0 | 0 | 0 | 1 | 1 | 0 | 0 | 0 | 0 | 0 | 0 | 0 | 0 | 3 | Original Literature |
| Cefdinir | 1 | 0 | 0 | 0 | 1 | 1 | 0 | 0 | 0 | 0 | 0 | 0 | 0 | 0 | 3 | Original Literature |
| Cefixime | 0 | 0 | 1 | 0 | 1 | 1 | 0 | 0 | 0 | 0 | 0 | 0 | 0 | 0 | 3 | Original Literature |
| Cefpodoxime | 1 | 0 | 0 | 0 | 1 | 1 | 0 | 0 | 0 | 0 | 0 | 0 | 0 | 0 | 3 | Original Literature |
| Rifampin | 1 | 0 | 0 | 0 | 1 | 0 | 0 | 0 | 0 | 1 | 0 | 0 | 0 | 0 | 3 | Original Literature |
| Azithromycin | 1 | 0 | 1 | 0 | 1 | 0 | 0 | 0 | 0 | 0 | 0 | 0 | 1 | 0 | 4 | Original Literature |
| Cefprozil | 1 | 0 | 1 | 0 | 1 | 1 | 0 | 0 | 0 | 0 | 0 | 0 | 0 | 0 | 4 | Original Literature |
| Ceftazidime | 0 | 0 | 1 | 0 | 1 | 1 | 0 | 0 | 1 | 0 | 0 | 0 | 0 | 0 | 4 | Original Literature |
| Cefuroxime | 1 | 0 | 1 | 0 | 1 | 1 | 0 | 0 | 0 | 0 | 0 | 0 | 0 | 0 | 4 | Original Literature |
| Chloramphenicol | 0 | 0 | 1 | 1 | 1 | 1 | 0 | 0 | 0 | 0 | 0 | 0 | 0 | 0 | 4 | Original Literature |
| Clarithromycin | 1 | 0 | 1 | 0 | 1 | 0 | 0 | 0 | 0 | 0 | 0 | 0 | 1 | 0 | 4 | Original Literature |
| Clindamycin | 1 | 0 | 1 | 1 | 0 | 0 | 0 | 0 | 0 | 1 | 0 | 0 | 0 | 0 | 4 | Original Literature |
| Piperacillin | 0 | 1 | 1 | 0 | 0 | 1 | 0 | 0 | 1 | 0 | 0 | 0 | 0 | 0 | 4 | Original Literature |
| Trimethoprim-sulfamethoxazole | 1 | 0 | 0 | 0 | 1 | 1 | 0 | 0 | 0 | 1 | 0 | 0 | 0 | 0 | 4 | Original Literature |
| Cefotaxime | 1 | 0 | 1 | 0 | 1 | 1 | 0 | 0 | 0 | 0 | 1 | 0 | 0 | 0 | 5 | Original Literature |
| Cefoxitin | 1 | 0 | 1 | 1 | 1 | 1 | 0 | 0 | 0 | 0 | 0 | 0 | 0 | 0 | 5 | Original Literature |
| Ceftriaxone | 1 | 0 | 1 | 0 | 1 | 1 | 0 | 0 | 0 | 0 | 1 | 0 | 0 | 0 | 5 | Original Literature |
| Colistimethate | 0 | 0 | 0 | 0 | 0 | 1 | 1 | 1 | 1 | 0 | 0 | 0 | 0 | 1 | 5 | Original Literature |
| Daptomycin | 1 | 1 | 0 | 0 | 0 | 0 | 0 | 0 | 0 | 1 | 0 | 1 | 0 | 1 | 5 | Original Literature |
| Doxycycline | 1 | 0 | 0 | 0 | 1 | 1 | 0 | 0 | 0 | 1 | 0 | 0 | 1 | 0 | 5 | Original Literature |
| Gentamicin | 1 | 0 | 0 | 0 | 1 | 1 | 0 | 0 | 1 | 1 | 0 | 0 | 0 | 0 | 5 | Original Literature |
| Minocycline | 1 | 0 | 0 | 0 | 1 | 1 | 0 | 0 | 0 | 1 | 0 | 0 | 1 | 0 | 5 | Original Literature |
| Telavancin | 1 | 1 | 0 | 0 | 0 | 0 | 0 | 0 | 0 | 1 | 1 | 0 | 0 | 1 | 5 | Original Literature |
| Tobramycin | 1 | 0 | 0 | 0 | 1 | 1 | 0 | 0 | 1 | 1 | 0 | 0 | 0 | 0 | 5 | Original Literature |
| Vancomycin | 1 | 1 | 0 | 0 | 0 | 0 | 0 | 0 | 0 | 1 | 1 | 0 | 0 | 1 | 5 | Original Literature |
| Amikacin sulfate | 1 | 0 | 0 | 0 | 1 | 1 | 0 | 0 | 1 | 1 | 0 | 0 | 0 | 1 | 6 | Original Literature |
| Amoxicillin-clavulanate | 1 | 1 | 1 | 1 | 1 | 1 | 0 | 0 | 0 | 0 | 0 | 0 | 0 | 0 | 6 | Original Literature |
| Ampicillin-sulbactam | 1 | 1 | 1 | 1 | 1 | 1 | 0 | 0 | 0 | 0 | 0 | 0 | 0 | 0 | 6 | Original Literature |
| Cefepime | 1 | 0 | 0 | 0 | 1 | 1 | 1 | 0 | 1 | 0 | 1 | 0 | 0 | 0 | 6 | Original Literature |
| Linezolid | 1 | 1 | 0 | 0 | 0 | 0 | 0 | 0 | 0 | 1 | 1 | 1 | 0 | 1 | 6 | Original Literature |
| Ticarcillin-clavulanate | 1 | 0 | 1 | 1 | 1 | 1 | 0 | 0 | 1 | 0 | 0 | 0 | 0 | 0 | 6 | Original Literature |
| Ceftaroline | 1 | 1 | 0 | 0 | 1 | 1 | 1 | 0 | 0 | 1 | 1 | 0 | 0 | 1 | 8 | Original Literature |
| Ciprofloxacin | 1 | 1 | 0 | 0 | 1 | 1 | 1 | 1 | 1 | 0 | 0 | 0 | 1 | 0 | 8 | Original Literature |
| Piperacillin-tazobactam | 1 | 1 | 1 | 1 | 1 | 1 | 1 | 0 | 1 | 0 | 0 | 0 | 0 | 0 | 8 | Original Literature |
| Ertapenem | 1 | 0 | 1 | 1 | 1 | 1 | 1 | 1 | 0 | 0 | 1 | 0 | 0 | 1 | 9 | Original Literature |
| Levofloxacin | 1 | 1 | 1 | 0 | 1 | 1 | 1 | 1 | 0 | 0 | 1 | 0 | 1 | 0 | 9 | Original Literature |
| Meropenem | 1 | 0 | 1 | 1 | 1 | 1 | 1 | 1 | 1 | 0 | 1 | 0 | 0 | 1 | 10 | Original Literature |
| Moxifloxacin | 1 | 1 | 1 | 1 | 1 | 1 | 1 | 0 | 0 | 1 | 1 | 0 | 1 | 0 | 10 | Original Literature |
| Imipenem-cilastatin | 1 | 1 | 1 | 1 | 1 | 1 | 1 | 1 | 1 | 0 | 1 | 0 | 0 | 1 | 11 | Original Literature |
| Tigecycline | 1 | 1 | 1 | 1 | 1 | 1 | 1 | 1 | 0 | 1 | 1 | 1 | 1 | 1 | 13 | Original Literature |
| Benzathine penicillin | 0 | 0 | 1 | 0 | 0 | 0 | 0 | 0 | 0 | 0 | 0 | 0 | 0 | 0 | 1 | Expert Panel Assignmen |
| Levornidazole | 0 | 0 | 1 | 1 | 0 | 0 | 0 | 0 | 0 | 0 | 0 | 0 | 0 | 0 | 2 | Expert Panel Assignmen |
| Cefaclor | 1 | 0 | 0 | 0 | 1 | 1 | 0 | 0 | 0 | 0 | 0 | 0 | 0 | 0 | 3 | Expert Panel Assignmen |
| Cefradine | 1 | 0 | 0 | 0 | 1 | 1 | 0 | 0 | 0 | 0 | 0 | 0 | 0 | 0 | 3 | Expert Panel Assignmen |
| Norfloxacin | 0 | 0 | 0 | 0 | 1 | 1 | 1 | 0 | 0 | 0 | 0 | 0 | 0 | 0 | 3 | Expert Panel Assignmen |
| Trimethoprim/Sulfamethoxazole | 1 | 0 | 0 | 0 | 1 | 1 | 0 | 0 | 0 | 1 | 0 | 0 | 0 | 0 | 4 | Expert Panel Assignmen |
| Colistin | 0 | 0 | 0 | 0 | 0 | 1 | 1 | 1 | 1 | 0 | 0 | 0 | 0 | 1 | 5 | Expert Panel Assignmen |
| Polymyxin B | 0 | 0 | 0 | 0 | 0 | 1 | 1 | 1 | 1 | 0 | 0 | 0 | 0 | 1 | 5 | Expert Panel Assignmen |
| Teicoplanin | 1 | 1 | 0 | 0 | 0 | 0 | 0 | 0 | 0 | 1 | 1 | 0 | 0 | 1 | 5 | Expert Panel Assignmen |
| Cefmetazole | 1 | 0 | 1 | 1 | 1 | 1 | 0 | 0 | 0 | 0 | 0 | 0 | 0 | 0 | 5 | Expert Panel Assignmen |
| Norvancomycin | 1 | 1 | 0 | 0 | 0 | 0 | 0 | 0 | 0 | 1 | 1 | 0 | 0 | 1 | 5 | Expert Panel Assignmen |
| Ceftazidime/Avibactam | 0 | 0 | 0 | 0 | 1 | 1 | 1 | 1 | 1 | 0 | 0 | 0 | 0 | 1 | 6 | Expert Panel Assignmen |
| Amoxicillin/Clavulanate | 1 | 1 | 1 | 1 | 1 | 1 | 0 | 0 | 0 | 0 | 0 | 0 | 0 | 0 | 6 | Expert Panel Assignmen |
| Faropenem | 1 | 0 | 1 | 1 | 1 | 1 | 0 | 0 | 0 | 0 | 1 | 0 | 0 | 0 | 6 | Expert Panel Assignmen |
| Cefminox | 1 | 0 | 1 | 1 | 1 | 1 | 1 | 0 | 0 | 0 | 0 | 0 | 0 | 0 | 6 | Expert Panel Assignmen |
| Contezolid | 1 | 1 | 0 | 0 | 0 | 0 | 0 | 0 | 0 | 1 | 1 | 1 | 0 | 1 | 6 | Expert Panel Assignmen |
| Ofloxacin | 1 | 0 | 0 | 0 | 1 | 1 | 1 | 0 | 1 | 0 | 1 | 0 | 1 | 0 | 7 | Expert Panel Assignmen |
| Isepamicin | 1 | 0 | 0 | 0 | 1 | 1 | 1 | 1 | 1 | 0 | 0 | 0 | 0 | 1 | 7 | Expert Panel Assignmen |
| Nitrofurantoin | 1 | 1 | 0 | 0 | 0 | 1 | 1 | 1 | 0 | 0 | 0 | 1 | 0 | 1 | 7 | Expert Panel Assignmen |
| Fosfomycin | 1 | 1 | 0 | 0 | 0 | 1 | 0 | 1 | 1 | 1 | 0 | 1 | 0 | 1 | 8 | Expert Panel Assignmen |
| Cefoperazone/Sulbactam | 1 | 0 | 1 | 1 | 1 | 1 | 1 | 1 | 1 | 0 | 0 | 0 | 0 | 1 | 9 | Expert Panel Assignmen |
| Sitafloxacin | 1 | 0 | 1 | 1 | 1 | 1 | 1 | 0 | 1 | 0 | 1 | 0 | 1 | 0 | 9 | Expert Panel Assignmen |
| Nemonoxacin | 1 | 1 | 0 | 0 | 1 | 1 | 1 | 0 | 0 | 1 | 1 | 1 | 1 | 1 | 10 | Expert Panel Assignmen |
| Eravacycline | 1 | 1 | 1 | 1 | 1 | 1 | 1 | 1 | 0 | 1 | 1 | 1 | 1 | 1 | 13 | Expert Panel Assignmen |
| Omadacycline | 1 | 1 | 1 | 1 | 1 | 1 | 1 | 1 | 0 | 1 | 1 | 1 | 1 | 1 | 13 | Expert Panel Assignmen |

Note: Scores for agents designated as 'Expert Panel Assignment' were determined by this study's specialists because these agents were not included in the original literature. The scoring was performed in a manner consistent with the methodology and rules established in the foundational paper (Gerber et al., 2017).

**Table S3.** **Covariate definitions**

| **Variable** | **Time Frame** | **Definition** | **Type** | **Analysis**  **format** |
| --- | --- | --- | --- | --- |
| **Sociodemographic characteristics** |  |  |  |  |
| Age | Time-fixed | Age at ICU admission | Continuous | years |
| Sex | Time-fixed | Patient's biological sex | Dichotomous | Male/Female |
| **Medical history** |  |  |  |  |
| Emergency Admission, n (%) | Time-fixed | Admission to ICU through the emergency department | Dichotomous | Yes/No |
| Prior hospitalization, n (%) | Time-fixed | Any hospitalization in the 2 months prior to the current hospitalization | Dichotomous | Yes/No |
| Prior antibiotic exposure, n (%) | Time-fixed | Use of any antibiotic within 30 days prior to hospital admission | Dichotomous | Yes/No |
| Prior antibiotic count, median (IQR) | Time-fixed | Number of distinct antibiotic classes used within 30 days prior to hospital admission | Continuous |  |
| Ward transfers, n (%) | Time-fixed | Number of transfers between hospital wards during the current hospitalization, prior to the first rectal swab screening | Continuous | Times |
| Previous ICU episodes in current stay, n (%) | Time-fixed | Number of ICU admissions during the current hospitalization, prior to the first rectal swab screening | Continuous | Times |
| surgery, n (%) | Time-fixed | Surgical procedure performed during the current hospitalization, prior to the first rectal swab screening | Dichotomous | Yes/No |
| **Comorbidities** |  |  |  |  |
| Renal failure | Time-fixed | Presence of pre-existing renal failure, defined by the presence of any of the following ICD-10 codes: I12.0, I13.1, N18, N19, N25.0, Z49.0, Z49.1, Z49.2, Z94.0, Z99.2, I120, I131, N18, N19, N250, Z490, Z491, Z492, Z940, Z992. | Dichotomous | Yes/No |
| Chronic pulmonary disease | Time-fixed | Presence of pre-existing chronic pulmonary disease, defined by the presence of any of the following ICD-10 codes (case-insensitive): I27.8, I27.9, J40, J41, J42, J43, J44, J45, J46, J47, J60, J61, J62, J63, J64, J65, J66, J67, J68.4, J70.1, J70.3, I278, I279, J40, J41, J42, J43, J44, J45, J46, J47, J60, J61, J62, J63, J64, J65, J66, J67, J684, J701, J703. | Dichotomous | Yes/No |
| Liver disease | Time-fixed | Presence of pre-existing liver disease, defined by the presence of any of the following ICD-10 codes: B18, I85, I86.4, I98.2, K70, K71.1, K71.3, K71.4, K71.5, K71.7, K72, K73, K74, K76.0, K76.2, K76.3, K76.4, K76.5, K76.6, K76.7, K76.8, K76.9, Z94.4, B18, I85, I864, I982, K70, K711, K713, K714, K715, K717, K72, K73, K74, K760, K762, K763, K764, K765, K766, K767, K768, K769, Z944. | Dichotomous | Yes/No |
| Cerebrovascular disease | Time-fixed | Presence of pre-existing cerebrovascular disease, defined by the presence of any of the following ICD-10 codes: G45, G46, H34.0, I60, I61, I62, I63, I64, I65, I66, I67, I68, I69, G45, G46, H340, I60, I61, I62, I63, I64, I65, I66, I67, I68, I69. | Dichotomous | Yes/No |
| Diabetes mellitus | Time-fixed | Presence of pre-existing diabetes mellitus, defined by the presence of any of the following ICD-10 codes (case-insensitive): E10, E11, E12, E13, E14, E100, E101, E102, E103, E104, E105, E106, E107, E108, E109, E110, E111, E112, E113, E114, E115, E116, E117, E118, E119, E120, E121, E122, E123, E124, E125, E126, E127, E128, E129, E130, E131, E132, E133, E134, E135, E136, E137, E138, E139, E140, E141, E142, E143, E144, E145, E146, E147, E148, E149. | Dichotomous | Yes/No |
| Elixhauser Comorbidity Index, median (IQR) | Time-fixed | A measure of comorbidity burden and illness severity, calculated based on the presence of 31 pre-defined comorbidities identified through ICD-10 diagnosis codes. | Continuous | Scores |
| SOFA score | Time-fixed | A measure of acute organ dysfunction and illness severity, calculated **within the first 24 hours of ICU admission** based on the degree of dysfunction across six organ systems (respiratory, cardiovascular, hepatic, coagulation, renal, and neurological). | Continuous | Scores |
| **Invasive devices use duration, n (%)** |  |  |  |  |
| Central venous catheter | Time-dependent | Refers to central venous catheter use within each screening period. | Dichotomous | Yes/No |
| Invasive mechanical ventilation | Time-dependent | Refers to invasive mechanical ventilation use within each screening period. | Dichotomous | Yes/No |
| Urinary catheter | Time-dependent | Refers to urinary catheter use within each screening period. | Dichotomous | Yes/No |
| Nasogastric tube | Time-dependent | Refers to nasogastric tube use within each screening period. | Dichotomous | Yes/No |
| Colonization pressure, median (IQR) | Time-dependent | For each screening period, colonization pressure was estimated as the average of the daily proportion of patients colonized/infected with CR-GNB. The daily proportion itself was calculated as the number of patients colonized or infected with CR-GNB divided by the total number of patients in the unit each day. | Continuous | % |
| **Other drugs, n (%)** |  |  |  |  |
| PPI or H_2_RA | Time-dependent | Use of Proton Pump Inhibitors or Histamine-2 Receptor Antagonists during each screening period. | Dichotomous | Yes/No |
| Corticosteroids | Time-dependent | Use of Corticosteroids during each screening period. | Dichotomous | Yes/No |

Abbreviations: CR-GNB, Carbapenem-resistant Gram-negative bacteria; H_2_RA, Histamine-2 Receptor Antagonist; ICD-10, International Classification of Diseases, Tenth Revision; ICU, Intensive Care Unit; IQR, Interquartile Range; SOFA, Sequential Organ Failure Assessment; PPI, Proton Pump Inhibitor.

**Table S4. Proportional hazards assumption testing results**

| Variable | Time-Varying Coefficient | | *P*-value |
| --- | --- | --- | --- |
|  | HR | 95% CI |  |
| ASI per antibiotic day | 0.999 | 0.996-1.003 | 0.787 |
| DDDs | 0.998 | 0.970-1.027 | 0.906 |
| LOT | 0.999 | 0.994-1.003 | 0.562 |
| Colonization pressure | 0.999 | 0.998-1.001 | 0.552 |
| Overall Test Results |  |  |  |
| Likelihood ratio test for PH assumption | χ^2^ = 0.963 |  | 0.915 |

Proportional hazards assumption was tested using time-varying coefficients. The likelihood ratio test comparing the standard Cox model with the time-varying coefficient model showed P = 0.915, indicating that the proportional hazards assumption was not violated. Abbreviations: ASI, Antibiotic Spectrum Index per antibiotic day; CI, Confidence Interval; DDDs, Defined Daily Doses; HR, Hazard Ratio; LOT, Length of Therapy.

**Table S5. Relative importance of variables**

| Variable | Chi-square | df | Importance | P-value |
| --- | --- | --- | --- | --- |
| ASI per antibiotic day | 26.92 | 1 | 25.92 | <0.001 |
| ICU Type | 11.36 | 2 | 9.36 | 0.045 |
| Colonization pressure | 8.73 | 1 | 7.73 | 0.003 |
| Renal failure | 4.28 | 1 | 3.28 | 0.039 |
| Ward transfers | 4.09 | 1 | 3.09 | 0.043 |
| SOFA score | 2.54 | 1 | 1.54 | 0.111 |
| LOT | 1.46 | 1 | 0.46 | 0.228 |
| Previous ICU episodes in current stay | 1.17 | 1 | 0.17 | 0.279 |
| Invasive mechanical ventilation | 1.02 | 1 | 0.02 | 0.312 |
| Age | 0.89 | 1 | -0.11 | 0.346 |
| Elixhauser Comorbidity Index | 0.79 | 1 | -0.21 | 0.376 |
| DDDs | 0.72 | 1 | -0.28 | 0.397 |
| Hospitalization in the one month | 0.61 | 1 | -0.39 | 0.435 |
| Corticosteroids | 0.54 | 1 | -0.46 | 0.461 |
| Nasogastric tube | 0.47 | 1 | -0.53 | 0.495 |
| Central venous catheter | 0.39 | 1 | -0.61 | 0.531 |
| Cerebrovascular disease | 0.22 | 1 | -0.78 | 0.638 |
| Surgery | 0.15 | 1 | -0.85 | 0.695 |
| PPI or H_2_RA | 0.1 | 1 | -0.9 | 0.75 |
| Gender | 0.08 | 1 | -0.92 | 0.783 |
| Urinary catheter | 0.06 | 1 | -0.94 | 0.806 |
| Chronic pulmonary disease | 0.05 | 1 | -0.95 | 0.827 |
| Diabetes mellitus | 0.05 | 1 | -0.95 | 0.82 |
| Emergency Admission | 0.03 | 1 | -0.97 | 0.86 |
| Liver disease | 0.03 | 1 | -0.97 | 0.853 |

Variables ranked by relative importance in predicting ICU-acquired CR-GNB in the fully adjusted interval-censored Cox regression model (Model 3). Importance was calculated as the partial Chi-square statistic minus the predictor's degrees of freedom (df). Abbreviations: ASI, Antibiotic Spectrum Index per antibiotic day; DDDs, Defined Daily Doses; df, degrees of freedom; H2RA, Histamine-2 Receptor Antagonist; ICU, Intensive Care Unit; LOT, Length of Therapy; PPI, Proton Pump Inhibitor; SOFA, Sequential Organ Failure Assessment.

**Table S6. Sensitivity Analyses**

| Sensitivity Analysis Scenario | N included | Adjusted HR (95% CI) | | |
| --- | --- | --- | --- | --- |
|  |  | ASI per antibiotic day | DDDs | LOT |
| Primary Analysis | 422 | 1.14 (1.09-1.19) | 0.89 (0.69-1.15) | 1.03 (0.97-1.11) |
| 1. Outcome restricted to CRE acquisition only | 345 | 1.13 (1.08-1.18) | 0.86 (0.66-1.12) | 1.03 (0.96-1.11) |
| 2. Standard Cox model used | 422 | 1.11 (1.07-1.15) | 1.14 (0.89-1.33) | 0.91 (0.78-1.03) |
| 3. Worst-case interval start for CRAB/CRPA | 422 | 1.07 (1.03-1.22) | 0.90(0.67-1.19) | 0.94 (0.80-1.07) |
| 4. Patients with prior 30-day antibiotic use excluded | 339 | 1.14 (1.07-1.41) | 1.15 (0.81-1.64) | 0.90(0.81-1.13) |
| 5.Subgroup: Outcome as Colonization Only | 398 | 1.15 (1.07-1.26) | 0.89 (0.73-1.21) | 1.02 (0.94-1.14) |
| 6.Subgroup: Outcome as Clinical Infection Only | 295 | 1.10 (1.02-1.33) | 1.12 (0.64-1.54) | 1.04 (0.78-1.16) |

Adjusted HR indicates Hazard Ratio from the fully adjusted interval-censored Cox regression model (corresponding to Model 3 in Table 2), controlling for demographics, comorbidities, invasive procedures, clinical variables, and the other two antibiotic exposure metrics. Abbreviations: ASI, Antibiotic Spectrum Index per antibiotic day; CI, Confidence Interval; CRAB, carbapenem-resistant Acinetobacter baumannii; CRE, carbapenem-resistant Enterobacterales; CRPA, carbapenem-resistant Pseudomonas aeruginosa; DDDs, Defined Daily Doses; HR, Hazard Ratio; LOT, Length of Therapy.


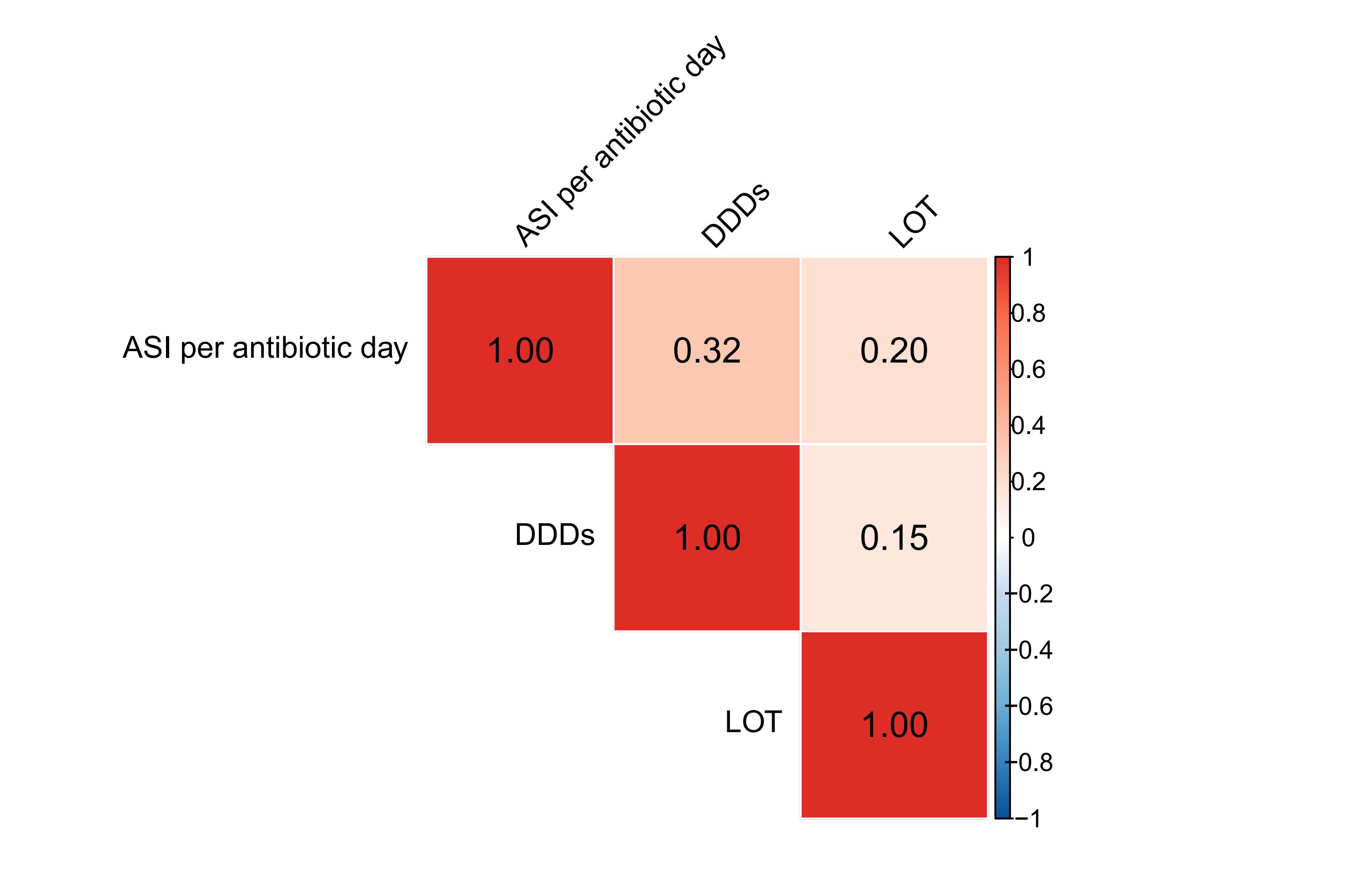


**Figure S1. Correlation matrix of antibiotic exposure metrics. The plot shows Pearson correlation coefficients between Antibiotic Spectrum Index (ASI), Defined Daily Doses (DDDs), and Length of Therapy (LOT). All correlation values were less than 0.4.**
